# Supplementary figures and images for: Cytomegalovirus late transcription factor target sequence diversity orchestrates viral early to late transcription
Source: PLoS Pathog. 2021 Aug 2;17(8):e1009796. doi: 10.1371/journal.ppat.1009796 (PMC8360532; doi:10.1371/journal.ppat.1009796)

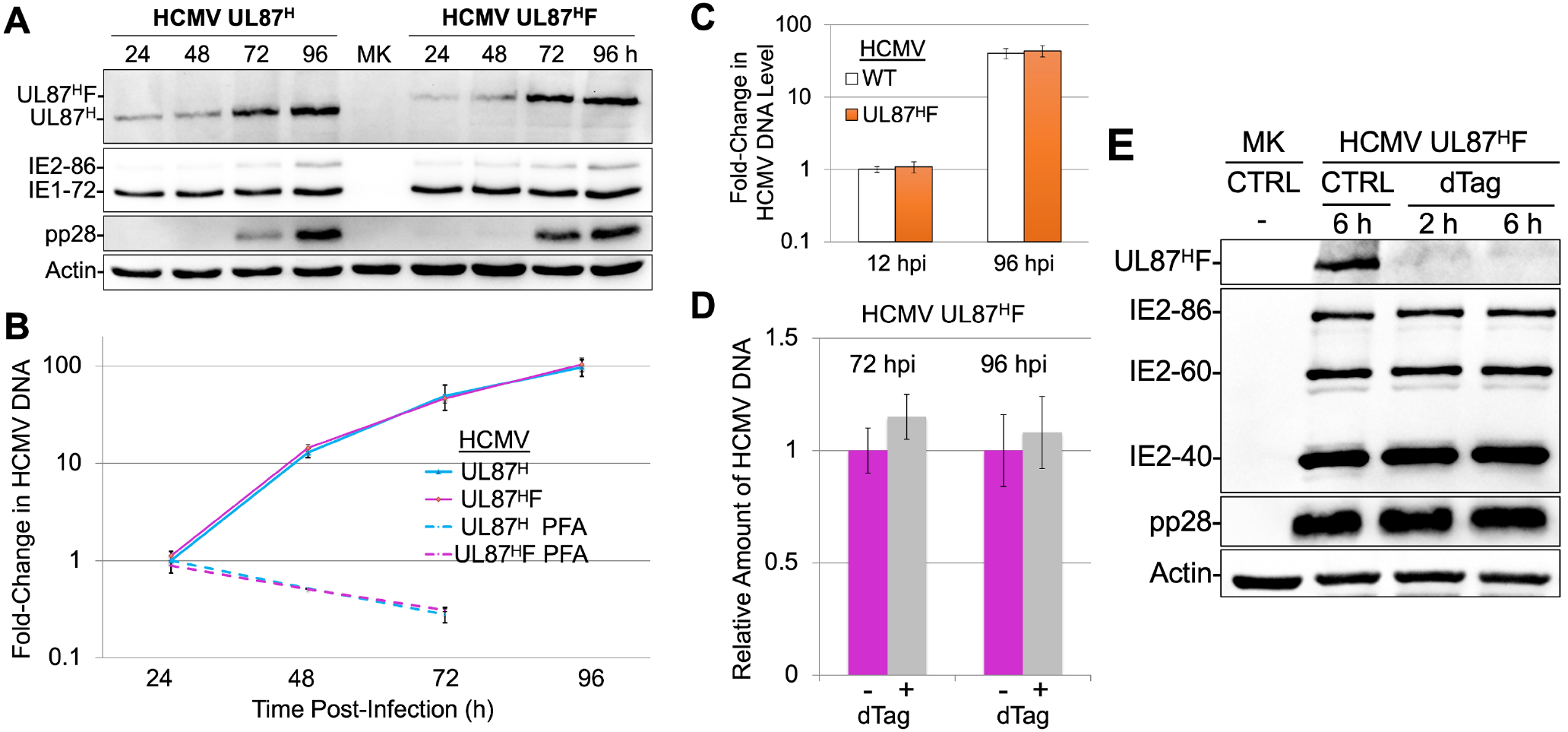

Supplement: S1 Fig — HCMV Towne UL87H and UL87HF at equivalent infectious units were applied to HFF. Whole cell lysates were analyzed at 24, 48, 72, and 96 hpi by (A) western blot (MOI of 3) with antibodies against the indicated viral and host proteins; and (B) qPCR (MOI of 0.5) to quantify HCMV genomes after normalization to host GAPDH DNA. Infections were also carried out for 72 h in presence of PFA to prevent viral DNA replication. Relative change in viral DNA level was computed using standard curve method; depicted as mean ±SD for 3 separate infections per group. (C) HCMV Towne WT and UL87HF DNA replication was analyzed at 12 and 96 hpi, according to method described in panel B. (D) The effect of 6-h dTag1 treatment on HCMV UL87HF DNA replication was measured by qPCR, using the method described above, and the results displayed relative to CTRL treatment (- dTag). The dTag1 (200 nM) was applied for the final 6 h of the 72 or 96 h infection. (E) dTag 1 (200 nM) was applied for the final 2 h and 6 h of the 96-h infection with HCMV UL87HF (MOI of 3). Vehicle (CTRL) was applied for the final 6 h. Western blot was performed with antibodies against UL87 HF, IE2-86, and late proteins IE2-40, IE2-60, and pp28. MK, mock infection. This is the same infection analyzed in Fig 7F. (TIF) [file ppat.1009796.s001.tif]

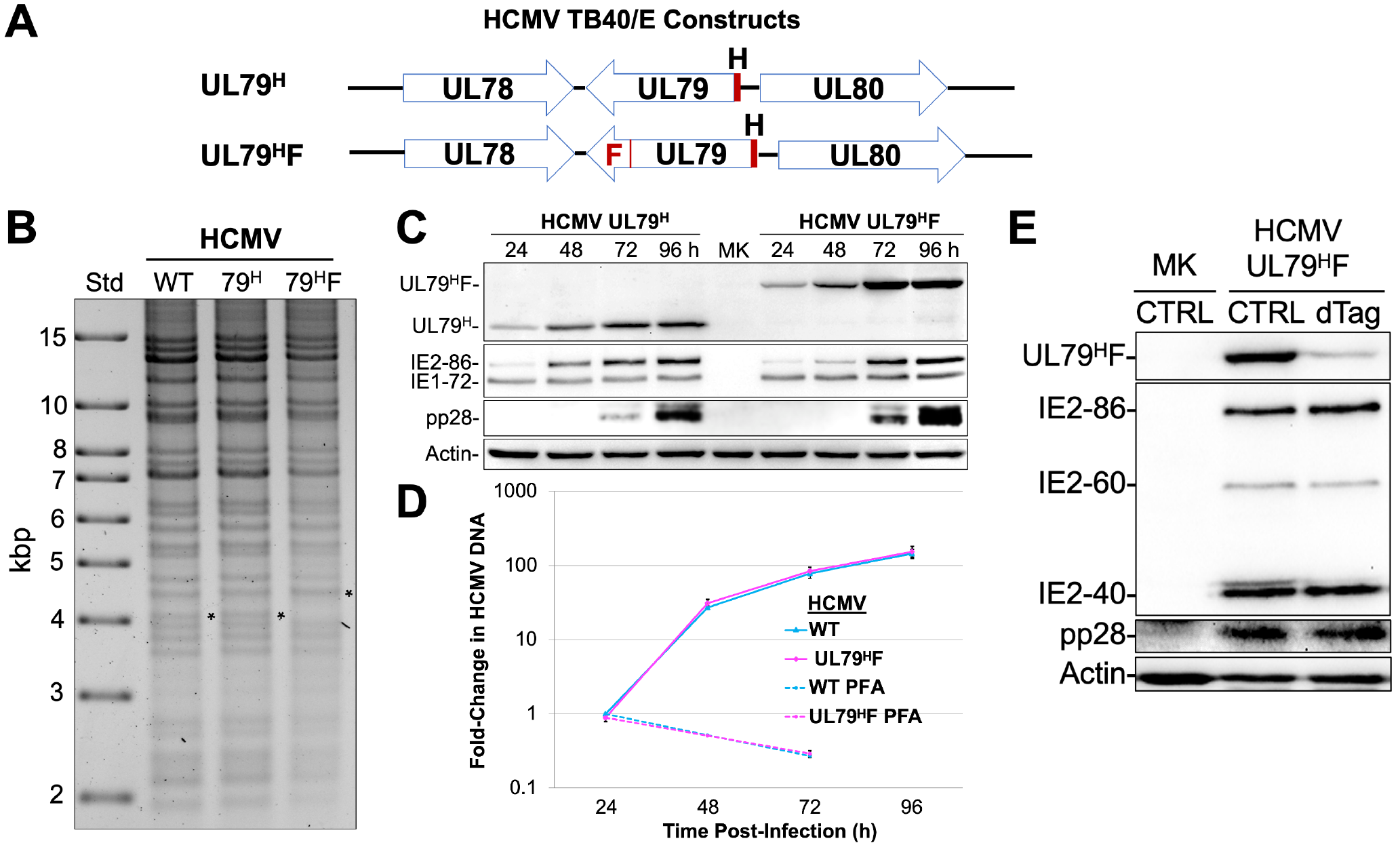

Supplement: S2 Fig — (A) Both viral constructs have an HA epitope (H) fused in frame to the amino end of the UL79 ORF. UL79HF has FKBP12 (F) fused in-frame to the carboxy end of the UL79 ORF. (B) Electrophoretic pattern of Bam HI fragments from genomes of the viral constructs and the parent WT virus. Astericks mark the restriction fragments containing the UL79 ORF. (C) Time course of tagged UL79 protein expression by UL79H and UL79HF in HFF at MOI of 3. The same western blot was re-probed for viral IE1, IE2, late protein pp28, and host actin. MK, mock infection. (D) Relative amount of UL79HF vs WT DNA at 24, 48, 72, and 96 h pi in HFF at MOI of 0.5. Results represent 3 biological replicates normalized to amount of host GAPDH DNA. PFA was present throughout the 72-h infection in a parallel set of infections. (E) The same protein extracts analyzed in Fig 1C were analyzed by western blot with antibodies against UL79HF, IE2-86, and late proteins IE2-40, IE2-60, and pp28. (TIF) [file ppat.1009796.s002.tif]

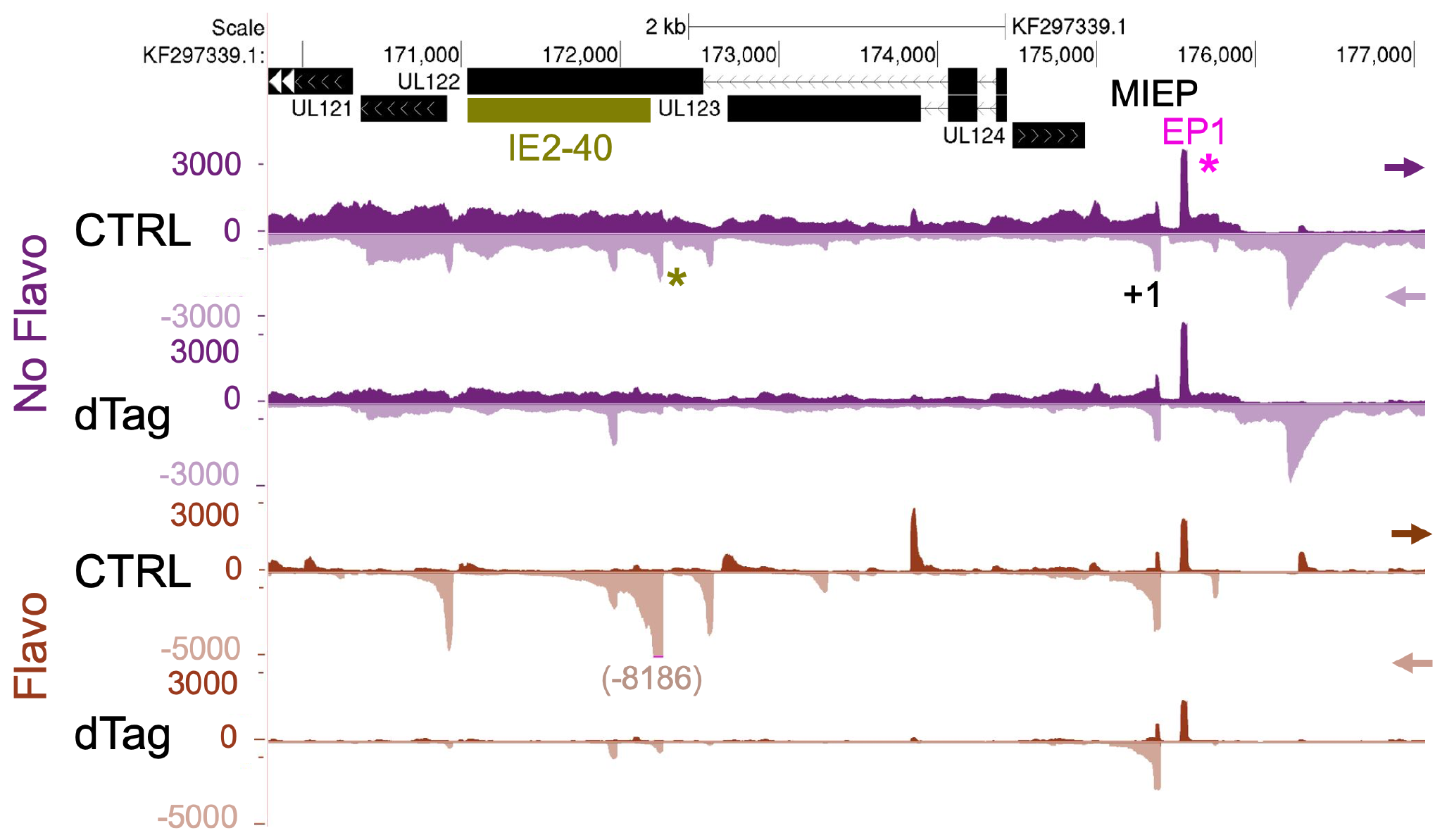

Supplement: S3 Fig — HFF infected with HCMV TB40/E UL79HF were treated with vehicle control (CTRL) or dTag-2 (dTag) for 6h at 66–72 hpi and Pol II nascent transcripts were then quantified by Pro-Seq minus and plus Flavo methods. Genome browser of reads aligned to the HCMV TB40/E KF297339 genome. Scale set at 3000 or 5000 reads to allow comparison of majority of viral TSRs. Track arrows point in direction of transcription. Asterisks mark TSRs for IE2-40 (olive green) and EP1 (pink). (TIF) [file ppat.1009796.s003.tif]

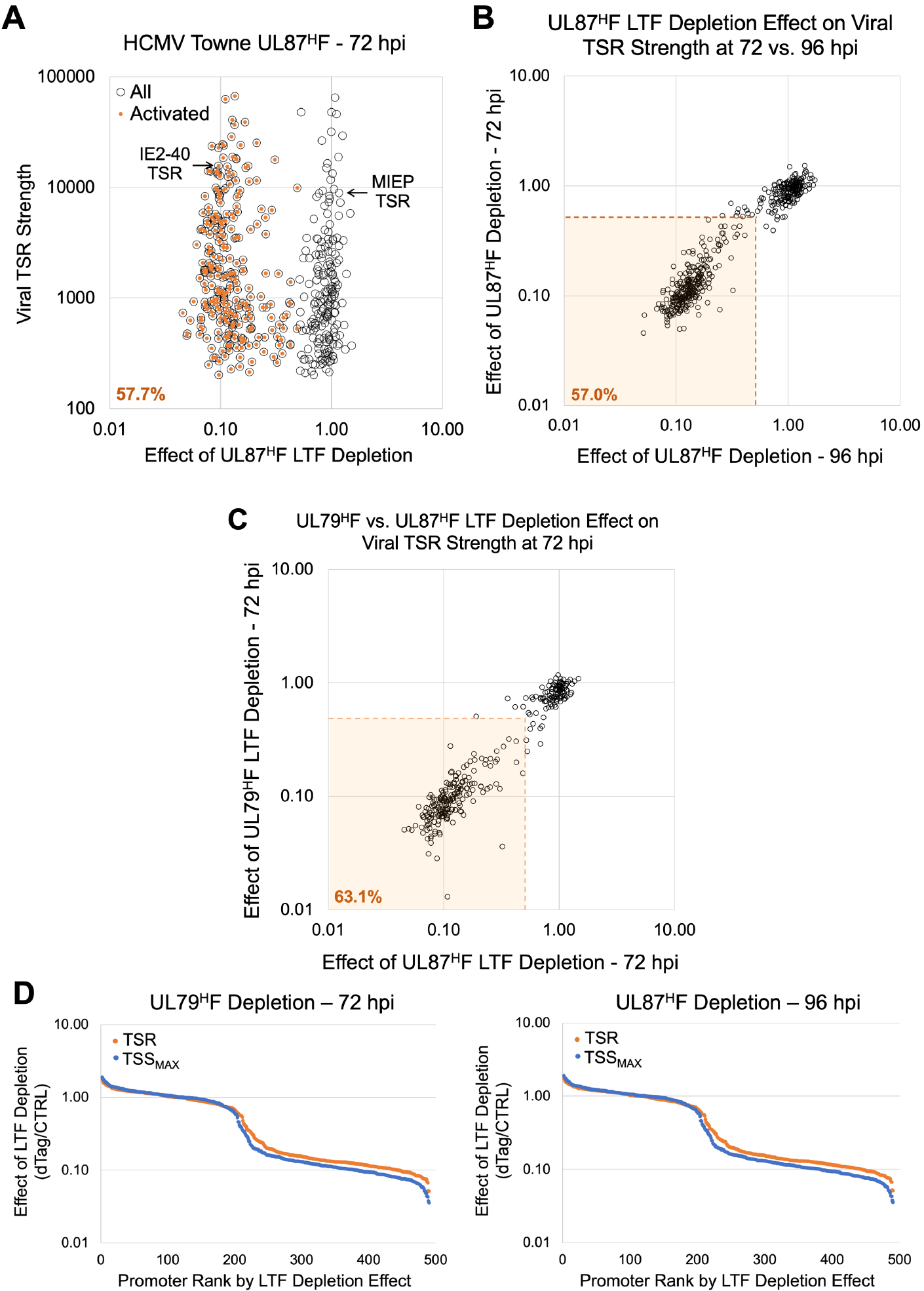

Supplement: S4 Fig — (A) Scatterplot of effect of UL87HF LTF depletion (6-h dTag treatment) vs. TSR strength at 72 hpi. Number of nascent RNA reads at each HCMV TSR (TSR strength) were quantified by PRO-Seq-Flavo method. Viral TSRs of >200 reads in CTRL group (open circles) were plotted against the change in TSR strength for dTag vs. CTRL treatment (dTag/CTRL). LTF-activated TSRs (orange dots) represent TSRs decreasing in strength by more than 50% because of dTag treatment. (B) Comparison of effects of depleting UL87HF LTF with dTag /CTRL treatment at 72 vs. 96 hpi on 461 viral TSRs (each TSR>200 CTRL reads), as measured by PRO-Seq-Flavo method. 266 TSRs at 72 hpi and 279 TSRs at 96 hpi decrease >50% because of UL87HF LTF depletion. Viral TSRs that decrease in strength by at least 50% at both time points (N = 263) are located in the gold shaded box with dashed borders and account for 57% of all viral TSRs. (C) Comparison of effects of depleting UL79HF vs. UL87HF LTF on viral TSRs (6-h dTag /CTRL) at 72 hpi. Viral TSRs conserved between Towne and TB40/E viruses (each TSR>200 CTRL reads) that decrease in strength by >50% (N = 188) are located in the gold shaded box with dashed borders (D) Effect of UL79HF or UL87HF depletion on viral MAXTSS and TSR strength for dTag vs. CTRL treatment (6-h dTag/CTRL). The MAXTSS for each viral TSR of >200 reads in CTRL group was included in the analysis. (TIF) [file ppat.1009796.s004.tif]

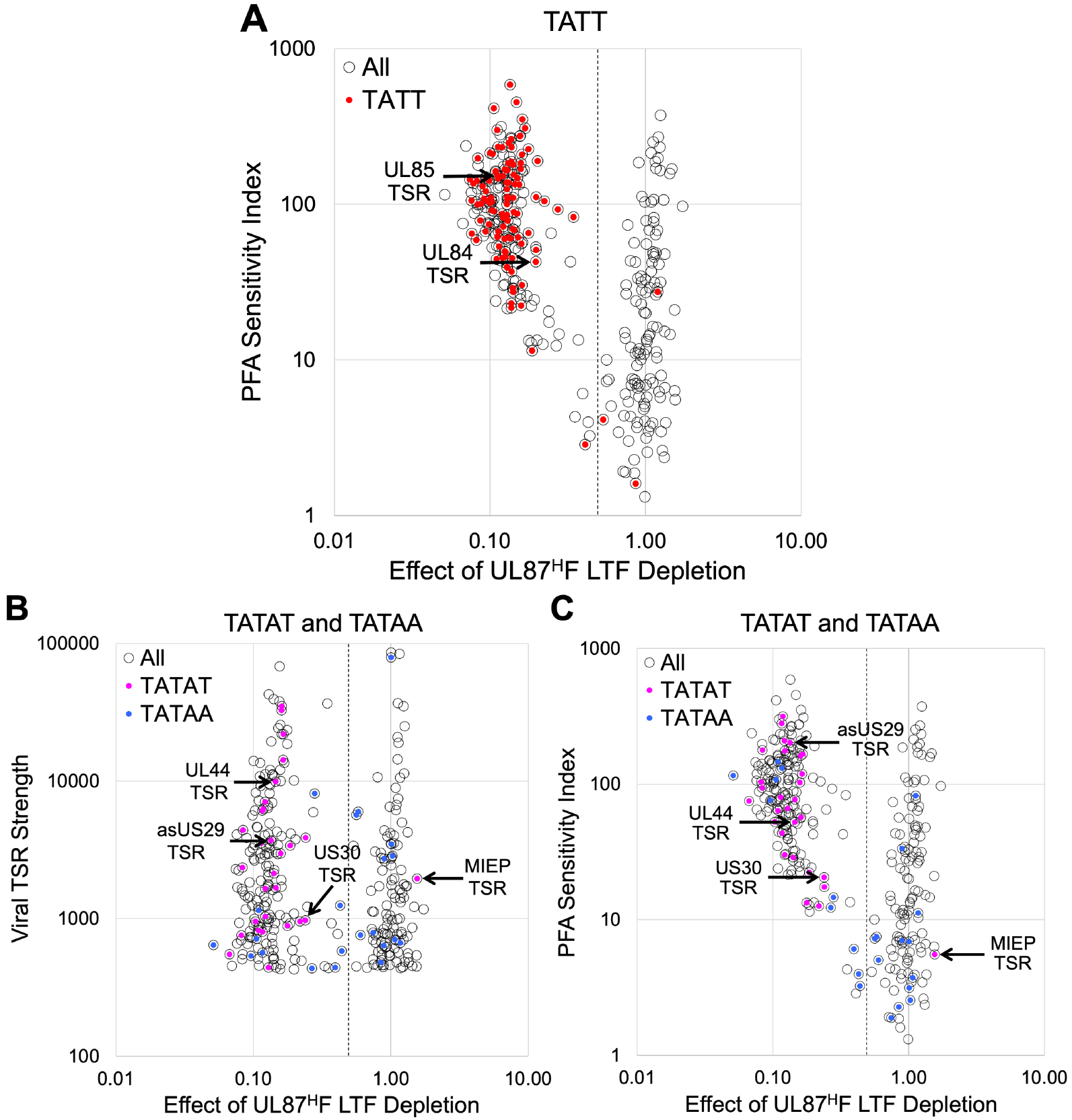

Supplement: S5 Fig — (A and C) Scatterplot of PSI vs. effect of UL87HF LTF depletion on TATT (A) and TATAT (C) promoters having TSR strength in top and mid tertiles. A set of 72-h Towne UL87HF infections were treated throughout with or without PFA. Effect of UL87HF LTF depletion was determined from infections described in Fig 3, in which dTag vs. CTRL was added for the last 6 h of infection. Spike-in normalized PRO-Seq-Flavo method was used to quantify change in viral TSRs. (B) Scatterplot of viral TSR strength vs. effect of UL87HF LTF depletion on TATAT promoters having TSR strength in top and mid tertiles. (TIF) [file ppat.1009796.s005.tif]

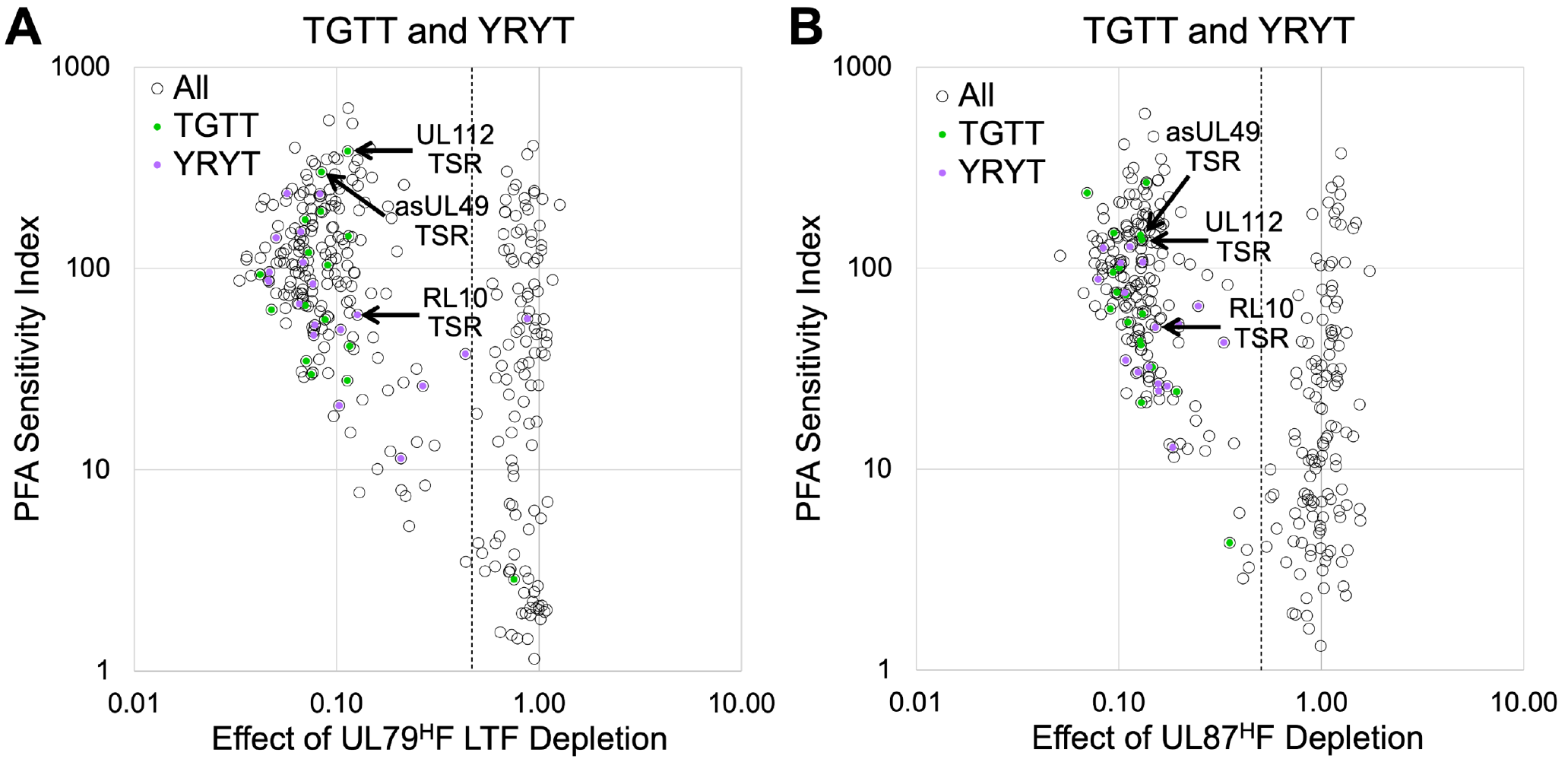

Supplement: S6 Fig — (A and B) Scatterplot of PSI vs. effect of UL79HF (A) and UL87HF (B) LTF depletion on TGTT and YRYT promoters having TSR strength in top and mid tertiles. Spike-in normalized PRO-Seq-Flavo method was used to quantify change in viral TSRs, as described in Fig 6 and S5 Fig. (TIF) [file ppat.1009796.s006.tif]
