# Supplementary material for: Cytomegalovirus late transcription factor target sequence diversity orchestrates viral early to late transcription
Source: PLoS Pathog. 2021 Aug 2;17(8):e1009796. doi: 10.1371/journal.ppat.1009796 (PMC8360532; doi:10.1371/journal.ppat.1009796)
Supplement: S1 Table — (PDF) [file ppat.1009796.s007.pdf]

TABLE S1. Nucleic Acid Reagents

A. Oligonucleotides and Gene Blocks

Oligo Name	Direction	Sequence (5’-to-3’)
**BAC Construction Oligos**		
TB40/E UL79N-**galK**	Forward	TGCTCGTCCATCGTCATTGTCCTCACCGTCGCTACCCGCTCACCGAGCGAACG**TCAGCACTGTCCTGCTCCTT**
TB40/E UL79N-**galK**	Reverse	TTGCCCGTGCGGACCCGCGGGACGGCGGGTTCTCTTCGTCGCGGGCCAT**CCTGTTGACAATTAATCATCGGCA**
TB40/E UL79C-**galK**	Forward	GGCCGTACTGGCCGCCCGCGCCCTTCATATGCCGACGCTGGCTAACGACGTG**TCAGCACTGTCCTGCTCCTT**
TB40/E UL79C-**galK**	Reverse	GCGCTGTACGGCAGCGGGGAGAAAAGTGGCAGATAAATCACGTCAGGTTCA**CCTGTTGACAATTAATCATCGGCA**
**Sequencing Oligos**		
TB40/E UL79N	Forward	GACTGGTCGTAGCGGGCGAG
	Reverse	CGCGGTGAAAGAGGAGGACGC
TB40/E UL79C	Forward	CCACAACAGCAGCAGCACCACCAC
	Reverse	TGTTTCGGCCGAAAGTTCCTCCGTCC
Towne UL87C	Forward	AAGATCAAGCACACTTGCGCGCTC
	Reverse	GCGGTAACACACGGCGAATCAGTT
**Gene Blocks (dsDNA)**		
TB40/E UL79N-**HA**		TGCTCGTCCATCGTCATTGTCCTCACCGTCGCTACCCGCTCACCGAGCGAACGATG**TACCCATACGATGTTCCAGATTACGCTCTG**ATGGCCCGCGACGAAGAGAACCCCGCCGTCCCGCGGTCCGCACGGGCAAATTC
TB40/E UL79C-**FKBP12**		GGCCGTACTGGCCGCCCGCGCCCTTCATATGCCGACGCTGGCTAACGACGTG**GGAGTGCAGGTGGAAACCATCTCCCCAGGAGACGGGCGCACCTTCCCCAAGCGCGGCCAGACCTGCGTGGTGCACTACACCGGGATGCTTGAAGATGGAAAGAAAGTTGATTCCTCCCGGGACAGAAACAAGCCCTTTAAGTTTATGCTAGGCAAGCAGGAGGTGATCCGAGGCTGGGAAGAAGGGGTTGCCCAGATGAGTGTGGGTCAGAGAGCCAAACTGACTATATCTCCAGATTATGCCTATGGTGCCACTGGGCACCCAGGCATCATCCCACCACATGCCACTCTCGTCTTCGATGTGGAGCTTCTAAAACTGGAA**TGAACCTGACGTGATTTATCTGCCACTTTTCTCCCCGCTGCCGTACAGCGCC

B. Adapters and Illumina index primers

**Adapters**
VRA3-8N: /5Phos/rNrNrNrNrNrNrNrNrGrArUrCrGrUrCrGrGrArCrUrGrUrArGrArArCrUrCrUrGrArArC/3InvdT/
VRA5-8N: rCrCrUrUrGrGrCrArCrCrCrGrArGrArArUrUrCrCrArNrNrNrNrNrNrNrN
**Illumina index primers**
RP1: AATGATACGGCGACCACCGAGATCTACACGTTCAGAGTTCTACAGTCCGA

RPI-1 [ATCACG]:<br>CAAGCAGAAGACGGCATACGAGATCGTGATGTGACTGGAGTTCCTTGGCACCCGAGAATTCCA
RPI-2 [CGATGT]:<br>CAAGCAGAAGACGGCATACGAGATACATCGGTGACTGGAGTTCCTTGGCACCCGAGAATTCCA
RPI-3 [TTAGGC]:<br>CAAGCAGAAGACGGCATACGAGATGCCTAAGTGACTGGAGTTCCTTGGCACCCGAGAATTCCA
RPI-4 [TGACCA]:<br>CAAGCAGAAGACGGCATACGAGATTGGTCAGTGACTGGAGTTCCTTGGCACCCGAGAATTCCA
RPI-5 [ACAGTG]:<br>CAAGCAGAAGACGGCATACGAGATCACTGTGTGACTGGAGTTCCTTGGCACCCGAGAATTCCA
RPI-6 [GCCAAT]:<br>CAAGCAGAAGACGGCATACGAGATATTGGCGTGACTGGAGTTCCTTGGCACCCGAGAATTCCA
RPI-7 [CAGATC]:<br>CAAGCAGAAGACGGCATACGAGATGATCTGGTGACTGGAGTTCCTTGGCACCCGAGAATTCCA
RPI-8 [ACTTGA]:<br>CAAGCAGAAGACGGCATACGAGATTCAAGTGTGACTGGAGTTCCTTGGCACCCGAGAATTCCA
RPI-9 [GATCAG]:<br>CAAGCAGAAGACGGCATACGAGATCTGATCGTGACTGGAGTTCCTTGGCACCCGAGAATTCCA
RPI-10 [TAGCTT]:<br>CAAGCAGAAGACGGCATACGAGATAAGCTAGTGACTGGAGTTCCTTGGCACCCGAGAATTCCA
RPI-11 [GGCTAC]:<br>CAAGCAGAAGACGGCATACGAGATGTAGCCGTGACTGGAGTTCCTTGGCACCCGAGAATTCCA
RPI-12 [CTTGTA]:<br>CAAGCAGAAGACGGCATACGAGATTACAAGGTGACTGGAGTTCCTTGGCACCCGAGAATTCCA
RPI-18 [GTCCGC]:<br>CAAGCAGAAGACGGCATACGAGATGCGGACGTGACTGGAGTTCCTTGGCACCCGAGAATTCA
RPI-19 [GTGAAA]:<br>CAAGCAGAAGACGGCATACGAGATTTTCACGTGACTGGAGTTCCTTGGCACCCGAGAATTCCA
RPI-22 [CGTACG]:<br>CAAGCAGAAGACGGCATACGAGATCGTACGGTGACTGGAGTTCCTTGGCACCCGAGAATTCCA
RPI-25 [ACTGAT]:<br>CAAGCAGAAGACGGCATACGAGATATCAGTGTGACTGGAGTTCCTTGGCACCCGAGAATTCCA
RPI-27 [ATTCCT]:<br>CAAGCAGAAGACGGCATACGAGATAGGAATGTGACTGGAGTTCCTTGGCACCCGAGAATTCCA
RPI-29 [CAACTA]:

CAAGCAGAAGACGGCATACGAGATTAGTTGGTGACTGGAGTTCCTTGGCACCCGAGAAT
TCCA
