# Supplementary material for: Cytomegalovirus late transcription factor target sequence diversity orchestrates viral early to late transcription
Source: PLoS Pathog. 2021 Aug 2;17(8):e1009796. doi: 10.1371/journal.ppat.1009796 (PMC8360532; doi:10.1371/journal.ppat.1009796)
Supplement: S2 Table — (PDF) [file ppat.1009796.s008.pdf]

TABLE S2. PRO-Seq and ChIP-Seq Datasets

A. *Scaling factors for Towne UL87^{H}F PRO-Seq datasets*

Exp1 datasets	Total deduplicated reads	Total read correction factor	Human mapped reads	CMV mapped reads	Spike-in mapped reads
Exp1 96 hpi Flavo	24023580	0.998587055	18851095	5007315	165170
Exp1 96 hpi Flavo 2 h dTAG	26937463	0.890567757	20449921	6276283	211259
Exp1 96 hpi Flavo 6 h dTAG	18217973	1.316811481	15019743	3057499	140731
Exp1 96 hpi Flavo 6 h PFA	26432633	0.907576479	17028368	9234949	169316
Exp1 96 hpi Flavo 2 h dTAG 6 h PFA	24336531	0.985745914	21952032	2166147	218352
**Exp1 datasets**	**Corrected human reads**	**Corrected CMV reads**	**Corrected spike-in reads**	**Spike-in correction factor**	**Final correction factor**
Exp1 96 hpi Flavo	18824459	5000240	164937	1.10	1.10
Exp1 96 hpi Flavo 2 h dTAG	18212040	5589455	188140	0.96	0.86
Exp1 96 hpi Flavo 6 h dTAG	19778170	4026150	185316	0.98	1.29
Exp1 96 hpi Flavo 6 h PFA	15454546	8381423	153667	1.18	1.07
Exp1 96 hpi Flavo 2 h dTAG 6 h PFA	21639126	2135271	215240	0.84	0.83
**Exp2 Flavo datasets**	**Total deduplicated reads**	**Total read correction factor**	**Human mapped reads**	**CMV mapped reads**	**Spike-in mapped reads**
Exp2 72 hpi Flavo	32394120	1.004790538	26143033	6006885	244202
Exp2 72 hpi Flavo 6 h dTAG	34827770	0.934579080	29698944	4863931	264895
Exp2 72 hpi Flavo 6 h PFA	34021716	0.956721444	27044218	6639954	337544
Exp2 72 hpi Flavo 6 h dTAG 6 h PFA	28953615	1.124187955	24659862	4037504	256249
**Exp2 Flavo datasets**	**Corrected human reads**	**Corrected CMV reads**	**Corrected spike-in reads**	**Spike-in correction factor**	**Final correction factor**
Exp2 72 hpi Flavo	26268272	6035661	245372	1.12	1.13
Exp2 72 hpi Flavo 6 h dTAG	27756012	4545728	247565	1.11	1.04
Exp2 72 hpi Flavo 6 h PFA	25873783	6352586	322936	0.85	0.82
Exp2 72 hpi Flavo 6 h dTAG 6 h PFA	27722320	4538913	288072	0.96	1.08
**Exp2 No Flavo datasets**	**Total deduplicated reads**	**Total read correction factor**	**Human mapped reads**	**CMV mapped reads**	**Spike-in mapped reads**
Exp2 72 hpi	30873452	1.074872612	20451064	10262890	159498
Exp2 72 hpi 6 h dTAG	35496604	0.934878953	23986562	11307455	202587
**Exp2 No Flavo datasets**	**Corrected human reads**	**Corrected CMV reads**	**Corrected spike-in reads**	**Spike-in correction factor**	**Final correction factor**

Exp2 72 hpi	21982289	11031299	171440	1.05	1.13
Exp2 72 hpi 6 h dTAG	22424532	10571102	189394	0.95	0.89
**Exp3 datasets**	**Total deduplicated reads**	**Total read correction factor**	**Human mapped reads**	**CMV mapped reads**	**Spike-in mapped reads**
Exp3 72 hpi Flavo	54772298	0.945175388	46600030	7108313	1063955
Exp3 72 hpi PFA Flavo	48766558	1.061576419	47466554	433037	866967
**Exp3 datasets**	**Corrected human reads**	**Corrected CMV reads**	**Corrected spike-in reads**	**Spike-in correction factor**	**Final correction factor**
Exp3 72 hpi Flavo	44045201	6718602	1005624	0.96	0.91
Exp3 72 hpi PFA Flavo	50389374	459702	920352	1.05	1.11

B. *Scaling factors for TB40/E UL79^{H}F and WT PRO-Seq datasets*

No Flavo datasets	Total deduplicated reads	Total read correction factor	Human mapped reads	CMV mapped reads	Spike-in mapped reads
Exp1 72 hpi UL79^{H}F	43312823	1.060794710	36709883	5851979	750961
Exp1 72 hpi UL79^{H}F dTAG	48579204	0.945795932	41802654	5725503	1051047
**No Flavo datasets**	**Corrected human reads**	**Corrected CMV reads**	**Corrected Spike-in reads**	**Spike-in correction factor**	**Final correction factor**
Exp1 72 hpi UL79^{H}F	38941650	6207748	796615	1.12	1.06
Exp1 72 hpi UL79^{H}F dTAG	39536780	5415157	994076	0.90	0.95
**Flavo datasets**	**Total deduplicated reads**	**Total read correction factor**	**Human mapped reads**	**CMV mapped reads**	**Spike-in mapped reads**
Exp1 72 hpi UL79^{H}F Flavo	54706702	1.031828824	49857798	3110425	1738479
Exp1 72 hpi UL79^{H}F Flavo dTAG	58189202	0.970076063	53856653	2182385	2150164
**Flavo datasets**	**Corrected human reads**	**Corrected CMV reads**	**Corrected spike-in reads**	**Spike-in correction factor**	**Final correction factor**
Exp1 72 hpi UL79^{H}F Flavo	51444713	3209426	1793813	1.08	1.12
Exp1 72 hpi UL79^{H}F Flavo dTAG	52245050	2117079	2085823	0.93	0.90
**Time course datasets**	**Total deduplicated reads**	**Total read correction factor**	**Human mapped reads**	**CMV mapped reads**	**Spike-in mapped reads**
Exp2 12 hpi WT Flavo	53088646	1.141981960	49677875	289499	3121272
Exp2 48 hpi WT Flavo	63422084	0.955917437	60358754	1093458	1969872
Exp2 48 hpi WT PFA Flavo	65368098	0.927459691	63501550	275996	1590552

Time course datasets	Corrected human reads	Corrected CMV reads	Corrected Spike-in reads	Spike-in correction factor	Final correction factor
Exp2 12 hpi WT Flavo	56731237	330603	3564436	0.65	0.74
Exp2 48 hpi WT Flavo	57697985	1045256	1883035	1.23	1.17
Exp2 48 hpi WT PFA Flavo	58895128	255975	1475173	1.56	1.45

C. *ChIP-Seq datasets*

ChIP-Seq datasets	Total deduplicated reads	Human mapped reads	CMV reads
Towne UL87^{H} HA	82846762	13306092	69540670
Towne UL87^{H} Pol II	68397980	36266593	32131387
TB40/E UL79^{H}F HA	28339209	8439843	19899366
TB40/E UL79^{H}F Pol II	30113611	20630155	9483456
