# Supplementary material for: Cytomegalovirus late transcription factor target sequence diversity orchestrates viral early to late transcription
Source: PLoS Pathog. 2021 Aug 2;17(8):e1009796. doi: 10.1371/journal.ppat.1009796 (PMC8360532; doi:10.1371/journal.ppat.1009796)
Supplement: Attachment — Submitted filename: Letter.pdf [file ppat.1009796.s010.pdf]

UNIVERSITY of IOWA HEALTH CARE

University of Iowa College of Medicine

Department of Internal Medicine
Division of Infectious Diseases
200 Hawkins Dr., SW34 GH
Iowa City, Iowa 52242-1009
319-356-7055 Phone
319-384-7208 ***Fax***

June 19, 2021

Dear Dr. Glaunsinger,

Thank you for serving as Guest Editor for our manuscript. We also thank the Editors for considering our manuscript for publication in PLOS Pathogens and are grateful to the reviewers for their thoroughness in critiquing the contents of this report and its conclusions. We have revised the manuscript to address all the requests below, as well as other recommendations or concerns expressed by reviewers. These changes have clearly improved the manuscript. We apologize for the delay in submitting the revised manuscript. The delay was not due to the requested changes.

Please note that the temporary Github links to view aligned reads using the USCS Genome Browser had to be changed. We've added a track to the TB40/E Github site to view the map of 5'-ends of reads at 48 hpi and a track to the Towne Github to view UL87 LTF ChIP-Seq Fragment Centers (explained below).

The new temporary Github links are:
https://github.com/meierjl/Towne-UL87LTF
https://github.com/meierjl/TB40E-UL79LTF

We have addressed each of your requests below, as well as the other comments or concerns of reviewers.

1) Describe differences in TSR usage between Towne and TB40/E.

We have provided information in an Excel file (**S1 Data**) that partly addresses this request. We had not designed experiments to directly compare Towne and TB40/E with respect to the strength of individual TSRs. This would require parallel infections at same MOIs, parallel sample processing and library construction, and the sequencing of these libraries on the same lane at the same depth. The aim of our study was to identify viral TSRs that are activated by LTFs and to determine if Towne and TB40/E differ in this regard. Fig. 3C and S1 Data addresses the later. The results section has been edited to reference S1 Data, and the paragraph below has been added to the Methods section.

"Comparison of HCMV Towne and TB40/E TSRs
The one-to-one comparison of TSRs of HCMV Towne BAC and TB40/E BAC viruses was performed on viral TSR measuring >200 reads in the CTRL-treated infections and conserved between the two viruses. Supplementary dataset 1 (**S1 Data**) lists each TSR >200 reads for CTRL-treated HCMV Towne BAC (72 and 96 hpi) and TB40/E BAC (72 hpi) viruses, according to TSR strength (number of reads) and TSR base position in Towne FJ616285 and TB40/E KF297339 genomes. The genome position for each Towne BAC virus TSR is listed alongside the genome position for the corresponding TB40/E BAC virus and vice versa. The dataset also lists the TSRs present in Towne BAC virus but not in TB40/E BAC virus and vice versa. Caution should be used in comparing viruses with

respect the strength of individual TSRs because Towne and TB40/E infections, sample processing, library construction, and sequencing were not performed in parallel."

2) Provide a figure comparing LTF ChIP-Seq signal and TSR strength.

We appreciate having been given the opportunity to complete what we had planned to do. A paragraph and figure (**Fig. 9A and B**) were added to the Results section to report a positive correlation between LTF ChIP-Seq signal and TSR strength (Spearman correlation coefficient, 0.733). To increase the signal-to-background noise ratio for measuring LTF ChIP-Seq peak size, we aligned the midpoint (center) of each UL87 LTF ChIP-Seq fragment of 100-200 bp length, termed Frag Center, to the HCMV Towne genome. A track was added to the Towne Github to view aligned Frag Centers vs. total fragments (18-400 bp length). LTF ChIP-Seq peak size was determined by counting Frag Centers located within a 200 bp window centered on base position -25 relative to the MAXTSS of the promoter's TSR. Each fragment read is unique (PCR duplicates are not included in the count) and its ends were determined from paired-end sequencing. We explain this approach in more detail in the revised results and methods sections.

3) Clarify your reason for comparing 72 hpi and 96 hpi.

We have added this information to the revised manuscript that provides a nice segue (below) into the paragraph that first describes the effect of UL79 LTF depletion on viral transcription. In reframing of the paragraph, we have added more information about the UL79 LTF depletion results at 72 hpi compared to UL87 LTF depletion results at 72 vs. 96 hpi. A new supplementary figure panel (**Fig., S4C**) was added for this purpose. The new supplementary dataset includes UL87 LTF depletion results for 72 and 96 hpi. The genome position for each Towne BAC virus TSR at 72 and 96 hpi is listed alongside the genome position for the corresponding TB40/E BAC virus.

Excerpts from the modified paragraph include the following:

"Our initial study of using dTag1 treatment to deplete the UL87 LTF was carried out at 96 hpi to allow comparison of results to those produced by dTag1-induced depletion of viral IE2 proteins at the same late infection timepoint [26]. In the subsequent study of dTag2-induced UL79 LTF depletion, PRO-Seq-Flavo was applied at 72 hpi to better compare our results to those generated by other investigators using different methods that were applied at this late infection timepoint [6]. The genome browser views of the effects of UL79 LTF depletion at 72 hpi resulting from 6-h dTag2 treatment (66-72 hpi) show changes in viral transcription that are remarkably like the changes observed at 96 hpi because of depleting UL87 LTF for 6 h."

"Based on findings of HCMV UL87^{H}F infections later carried out for 72 h using a different viral stock and HFF line, we determined the 1-day difference in time of late infection does not substantially affect the overall weight of results. Only 2.5% fewer viral TSRs decrease >50% in strength at 72 hpi compared to 96 hpi when depleting the UL87 LTF for the last 6 h of these infections (**Fig. S4A and B**). Cross-comparing HCMV Towne UL87 LTF depletion results with those from the TB40/E UL79 LTF depletion revealed that the UL87 LTF depletions resulted in 1.5% and 2.6% more viral TSRs decreasing >50% at 72 and 96 hpi, respectively (**Fig. S4C and S1 Data**), which is probably because UL87 LTF depletion is slightly more robust than that of UL79 LTF depletion (**Fig. 1**)."

4) *Temper your conclusion that the octanucleotide block is the sole regulator of promoter strength.*

We had not realized that our writing would result in the perception that the octanucleotide block is the sole regulator of promoter strength. Our study approach enabled us to apply statistical methods to describe the strength of the relationship between sequence code in the octanucleotide block and functional outcome. The conclusion that sequence code in the octanucleotide block predicts functional outcome is supported by results from the MEME logo prediction tool applied according to tertile rank in promoter strength. We were careful to not assert that we have proven causality. We have edited the discussion to clarify that sequence code in the oligonucleotide block is not the sole determinant of promoter strength. We have also edited the author summary by changing the sentence "Diversity in signature patterns expands promoter targets and pre-sets amount of individual promoter output" to diversity in signature patterns expands promoter targets and *probably* pre-sets amount of individual promoter output". We also edited Introduction to say, "…contribution of HCMV LTF target sequence diversity in expanding the pool of LTF targets and *likely* determining individual promoter output."

We wish to point out that we had provided reasons in the results section that led us to previously conclude that "Two promoters having the same 10-base TATTAAAGGT motif positioned -31 from the MAXTSS but differing 12-fold in TSR strength (2744 vs 224 reads) adds to the notion that other sequences or factors are contributing to TSR strength" (line 311 in marked up manuscript) and "We have not discounted the possibility that nucleotide code in flanking sequence is a factor that modifies TSR strength." (line 319 in marked up manuscript). In the discussion we also had stated "We surmise that the LTF target sequence code determines probability of promoter activity based on several lines of circumstantial evidence." (line 549 in marked up manuscript).

We have edited a paragraph in the Discussion to state the octanucleotide code is not the sole determinate of promoter strength. We had previously stated that "Of the 21 LTF-driven promoters with a TATTAAA, only half of them rank in the top tertile of promoter strength and this top half appears to have G or C enrichment immediately downstream of the TATTAAA". This is changed to: "Of the 21 LTF-driven promoters with a TATTAAA, only half of them rank in the top tertile of promoter strength and this top half appears to have G or C enrichment in downstream flanking sequence. This observation indicates that sequence code in the octanucleotide block is not the sole determinant of promoter strength." We also edited another sentence in the paragraph to say: "TATTW variants that are not TATTWAA account for 24% of top performing promoters and generally *but not always* conform to the TATTWRMS".

5) *Describe how you performed the calculation of promoter activity on a given genome*.

We have revised our previous version of the message that we had inadequately conveyed with the following:

"Our study methodology provides a snapshot in time of the quantity of Pol II nascent RNA reads at a promoter's MAXTSS (or TSR) that represents the frequency with which Pol II is engaged at the promoter among a population of genomes carrying this promoter. Analysis of PRO-Seq-Flavo findings for the infection condition shown in Figure 2A indicates that ~1 million HCMV genomes have Pol II engaged at the RNA4.9 promoter at 96 hpi. If we venture the assumption that 1 million viral genomes are amenable to Pol II transcription at other viral promoters, it will take 0.1-3.0% of such viral genomes at that

point in time for Pol II engagement to yield TSR strength values of the top performing LTF-responsive promoters described in this report. An LTF-responsive promoter falling in the bottom tertile of TSR strength would be active on <0.03% of such viral genomes."

6) Modify Figures 6B and 8 to make scales the same and easier for cross comparison.

Figure 6B (and legend) has been changed to make scales the same at each timepoint. To improve cross comparison, we also changed panel C. We had previously mapped 5'-ends of PRO-Seq reads for 12 and 72 hpi. We thought it was best to instead show the 5'-end maps of the aligned viral reads shown in panel B for 12 and 48 hpi. The Genome Browser track for the 5'-end map at 48 hpi has been added to the Github site. Because viral DNA amplification greatly increases the number of viral reads, the scale at 48 hpi differs from that of 12 hpi (pre-DNA replication).

Figure 8 is scaled to show examples of LTF-responsive promoters that have variant sequences that are bound by LTFs. If we scale both forward and reverse tracks the same, some of the viral TSRs of interest will not be viewable. TSRs of lower strength are often on the strand opposite to that having a stronger TSR in the neighborhood. For example, the bidirectional promoter yields TSRs that differ by 11-fold in strength, as described in the Results section. The Results section has been modified, with addition of a new Fig. 9, to provide an appraisal of the potential relationship between LTF ChIP-Seq signal and viral TSR strength.

We have edited Figure 8 to now include read numbers for ChIP-Se tracks. These were previously omitted to fit figure panels onto a single page. We have corrected this error by re-sizing figure panels. We also labeled the Y-axis of the heatmap of ChIP-Seq fragments according to fragment length.

Reviewer 1.

1a) The conclusion that diversity in LTF target sequences shapes the LTF-dependent program that drives the viral early-late transcription switch is not novel and had been reported multiple times.

We are pleased that the reviewer appreciates the importance of this topic and the potential role LTF target sequence diversity in determining promoter strength. We are puzzled by the comment that diversity in LTF target sequences is a widely held view that has been published multiple times. In our PubMed search, we had not come across publications that have addressed the diversity issue particularly in the context of the viral genome in infected cells. It is possible we have inadvertently overlooked such a publication, since we were not provided with examples of publications that were omitted and/or should have been addressed. We agree that it is well known that LTF target sequences are important in driving the viral early-late transcription switch.

From our studies, we have 1) identified previously unrecognized TATT-less target sequences, 2) exposed the unanticipated variety in LTF target sequence options, 3) determined the wide range (orders of magnitude) in strength of LTF-driven promoter transcription, 4) described target sequence signatures that predict strong vs. weak promoter transcription, and 5) ascertained the extent to which LTF-dependent promoters regulate early-late kinetic-class promoters, in addition to the classical true late promoters.

1b) Lack of strong evidence that sequence diversity in the octanucleotide block is the sole regulator of promoter strength.

We have addressed this issue above.

1c) No experiments were performed to assess functional importance of diversity in the octanucleotide block and flanking sequences.

Strong associations do not prove causality. We agree that future studies will be required to prove causality. This best be done by carefully engineering site-directed mutations in the viral genome.

2) Need to compare LTF ChIP-Seq signal and TSR strength.

This issue has been addressed above and the manuscript has been revised accordingly.

3) Why were 72 hpi vs 96 hpi timepoints used in comparison of UL79 and UL87 LTF depletions?

This issue has been addressed above and manuscript revised accordingly. The difference between 72 and 96 hpi for UL87 LTF depletion results are minimal (**Fig. S4**) and do not change conclusions.

4) Need for additional*information on the effect of LTF depletion on late protein expression*.

This information was added to Figs S1 and S2 (panels E)

5) Fig 3 legend wording confusing. Panels A and B need cutoff line that denotes 0.5 value. Why the differential sensitive of LTF-promoters to LTF depletion?

Figure 3 legend wording 'LTF-activated' changed to LTF-responsive. Legend has been changed to explain hatched breakpoint lines in panels A and B. We do not know the answer to the question. One possibility is that LTF and TBP preinitiation complexes may compete for some viral promoters.

6) Why are 4 TATT promoters unresponsive to LTFs?

Good question. We do not have an answer to the question. As had been mentioned in the results section (lines 370 and 371 in marked up manuscript), 2 of these 4 TATT promoters are controlled by viral IE2 in late infection.

New discoveries raise new questions and the depth of these studies identified outliers that are nonconformists. The MIE promoter is an example of an elephant in the room given its LTF-independent performance while fitted with a TATAT motif as its TATA box that is a LTF recognition site not bound by LTF.

7) Adjust Fig. 6 and 8 scales to assist in cross-comparison.

We have made changes to these figures as explained above.

8) No discussion regarding bidirectional promoters in Discussion section.

We had discussed bidirectional promoters toward the end of the discussion section, which is lines 570-574 in the revised marked up manuscript.

9) Consider moving lines 187-194 to the discussion.

We have decided to keep this information upfront to give the reader enough information about the PRO-Seq-Flavo approach to be able to judge our interpretation of results going forward.

Reviewer 2.

1) Carefully conducted study that yields important information but is a very dense, mostly characterization study.

We appreciate the reviewer's comments about the quality and novelty of this study. We agree with the assessment that our discoveries largely come from a deep comprehensive characterization of LTF target sequences in relation to temporal change and magnitude of promoter function. The integration of dTAG system, PRO-Seq, and ChIP-Seq provides new mechanistic insight into how UL79 and UL87 LTFs work. PRO-Seq method (plus or minus Flavo) reports on the LTF's regulatory actions in determining the frequency with which Pol II (with its nascent RNA) is engaged at the promoter or gene body among a population of genomes carrying this promoter and gene. For example, it was this information that revealed that UL79 LTF functions in transcription initiation rather than in productive elongation, as previously reported. Finding a good balance in presenting enough salient findings curated from large datasets is challenging. We believe we have met the challenge in providing sufficient detail for others to use in forwarding their studies of LTF biology and viral gene regulation.

Reviewer 3.

1) The authors do not compare TSR usage between Towne and TB40/E strains.

We have addressed this issue above and revised the manuscript accordingly.

2) Unproven claim that the differential expression of UL1 by Towne vs. TB40/E is due to a nucleotide change. Was there a difference in LTF occupancy?

We had not intended to claim a causal relationship but rather report a striking observation of an association. As the reviewer points out, the idea that change of the TATTTAA to TAcTTAA accounts for ~70-fold reduction in TSR strength is plausible. We have edited the sentence in the Results section by replacing the word inferred to suggested.

"The functional importance of the TATTW core is *suggested* by the observation that TSR strength decreases by almost 70-fold in association with a naturally occurring base-substitution in TATTTAA of the UL1 gene promoter in the Towne strain genome that converts this sequence to TATcTAA in the UL1 promoter in TB40/E strain genome."

Unfavorable LTF ChIP-Seq signal-to-background ratio in the genome region carrying the UL1 promoter did not permit a determination of whether there was difference in LTF binding to Towne vs. TB40/E UL1 promoter. The ChIP-Seq signal in this genome regions is viewable using the Github links provided.

3) Need to determine if LTF ChIP-Seq signal correlates with TSR strength.

As noted above, we have addressed this issue in considerable depth and revised the manuscript accordingly.

4) Were UL79 and UL87 LTFs associated with host promoters?

We looked but did not find evidence of LTFs occupying host promoters. We did not address this issue in the manuscript because we had not completed a rigorous study to prove a negative result that could be due to a variety of reasons.

5) Figure 4 legend does not clearly indicate which datasets were analyzed. Legend for Figure 3C does not state time after infection.

We apologize for having fallen short on describing the datasets used for host analysis. We have corrected this deficiency. All host analyses were done using 72 hpi datasets for which there were results of viral analyses displayed in other figures.

The legend for Fig. 3C scatterplot had time after infection in X and Y axis labels. We have added this information to legend as well.

6) *Why were 72 hpi vs 96 hpi timepoints used in comparison of UL79 and UL87 LTF depletions?*

This issue has been addressed above and manuscript revised accordingly. The difference between 72 and 96 hpi for UL87 LTF depletion results are minimal (**Fig. S4**) and do not change conclusions.

7) *Why are host promoters not susceptible LTF-mediated control?*

We have previously reported that LTFs do not control host promoters, based on findings produced by a different analytical approach (PMID: **30755505). Circumstantial evidence has led us to** propose that host chromatin structure makes host DNA inaccessible to LTFs. The findings in this report supports that hypothesis. We have added a sentence to discussion, "We have speculated that viral LTFs are unable to access the DNA in host chromatin [29]."

8) Unclear how the percentages of viral genomes supporting strong vs. weak LTF-dependent promoters were computed?

We have addressed this above and revised the paragraph to better explain how we arrived at these numbers.

Thank you for considering our revised manuscript for publication in PLOS Pathogens.

Sincerely,

Jeff Meier, MD
University of Iowa Carver College of Medicine
Iowa City Veterans Affairs Healthcare System
